# The Root Aqueous Extract of* Entada africana* Guill. et Perr. (Mimosaceae) Inhibits Implant Growth, Alleviates Dysmenorrhea, and Restores Ovarian Dynamic in a Rat Model of Endometriosis

**DOI:** 10.1155/2017/8563909

**Published:** 2017-12-31

**Authors:** Marie Alfrede Mvondo, Stéphane Minko Essono, Francis Désiré Bomba Tatsinkou, Sylvin Benjamin Ateba, Dieudonné Njamen

**Affiliations:** ^1^Laboratory of Animal Physiology and Phytopharmacology, Department of Animal Biology, Faculty of Science, University of Dschang, P.O. Box 67, Dschang, Cameroon; ^2^Laboratory of Animal Physiology, Department of Animal Biology and Physiology, Faculty of Science, University of Yaounde I, P.O. Box 812, Yaounde, Cameroon

## Abstract

*Entada africana *(Mimosaceae) was reported to have analgesic and antioxidant properties. The present study is aimed at investigating the effects of the root aqueous extract of* Entada africana *(EA) on an experimental model of endometriosis. The study was performed in rats orally treated with EA at doses of 127.5, 255, and 510 mg/kg. Microgynon® 30 served as the reference substance. Estradiol valerate and oxytocin were used to induce dysmenorrhea. Endometrial implant levels of catalase and malondialdehyde (MDA) allowed estimating tissue oxidative status. Ovarian dynamic and rat sexual behavior were assessed through histological analysis of ovaries, uterus, and vagina. EA decreased dysmenorrhea at tested doses following a 7-day treatment (*p* < 0.001). Endometrial implant volume decreased following the three treatment periods (*p* < 0.05). Catalase activity (*p* < 0.001) and MDA level (*p* < 0.01) increased only following a 3-day treatment. EA also increased antral follicles, reduced luteinized unruptured follicle number (*p* < 0.001), and induced animals to be in the estrus phase. In conclusion, EA prevented the progress of endometriosis, reduced dysmenorrhea, promoted ovarian follicle growth, prevented anovulation, and stimulated the special period of rat sexual desire. These results suggest that* Entada africana* could be a promising alternative option for the treatment of endometriosis.

## 1. Introduction

Endometriosis is a chronic estrogen-dependent condition that causes dysmenorrhea, nonmenstrual pelvic pain, dyspareunia, and infertility [1]. Due to its pain symptoms and high recurrence rate, endometriosis is often associated with severely altered quality of patients' private and professional life [2]. The treatment options are diverse and consist of analgesic therapies, hormonal therapies that focus on reducing systemic levels of estrogen (e.g., progestins, androgens, gonadotropin-releasing hormone (GnRH) agonists, oral contraceptives, and aromatase inhibitors), surgery, or a combination of these [3]. Unfortunately, these treatments are not fully effective and are associated with substantial side effects and frequent recurrences [4], hence the urgency to develop innovative active substances that are better tolerated and more efficient than the currently applied pharmacological and surgical approaches.

There are scientific evidences to suggest that medicinal herbs with analgesic and fertilizing properties are potential alternatives to treat endometriosis [5, 6].* Entada africana* Guill. et Perr. (Mimosaceae) for instance is an African medicinal plant traditionally used to treat various ailments including female infertility, malaria, and rheumatism [7, 8]. The root bark decoction of this plant is recommended in Tchabal (Adamaoua region, Cameroon) as a cure for several complaints including lower abdominal pain (Table 1). Moreover this plant has been subject to numerous scientific investigations reporting analgesic [9], anti-inflammatory [8, 9], and antioxidant [10] properties. Apigenin, a flavonoid isolated from the roots of* Entada africana* [11], was reported to display antioxidant activity [12], to inhibit cell proliferation [13, 14], and to have anti-inflammatory activity [15]. The presence of this compound in* Entada africana* roots may account for its scientific proven properties [8–10]. Although bearing all these properties,* Entada africana* has not yet been investigated for endometriosis and its related pain and ovarian dysfunction. The present study therefore is aimed at investigating the effects of the root aqueous extract of* Entada africana* in different doses and different treatment regimens, on endometriosis related dysmenorrhea and ovarian dynamic (this refers to follicle development, ovulation, formation, and regression of the corpora lutea) in an experimental rat model. Dysmenorrhea was evaluated through abdominal writhing response, and ovarian dynamic was evaluated through the modulation of the number of antral follicles, luteinized unruptured follicles (these are mature ovarian follicles that fail to rupture and undergo luteinization with trapped oocytes [16]), and corpora lutea. Rat sexual behavior was estimated through histological analysis of the uterus and the vagina.

## 2. Material and Methods

### 2.1. Plant Material and Preparation of the Aqueous Extract

The roots of* Entada africana *were collected in April 2016 in Tchabal (Cameroon Adamaoua region) where it is known as «Padde wandu» in Foulfouldé, «Wéléon» in Toupouri, or «Missi siriwé» in Guisiga, three Cameroonian vernacular languages. The plant was identified and authenticated in comparison with the botanical sample of C. Geerling N° 5188 registered at the Cameroon National Herbarium, where a voucher specimen has been deposited under the number 36694/HNC.

To carry out the present study, a small ethnobotanical survey was conducted in Tchabal (Cameroon Adamaoua region) in order to gain insight into the traditional uses of* Entada africana*, which allowed us to establish an appropriate extraction protocol, to determine the doses to be tested, and to define treatment periods. This ethnobotanical survey was conducted using a structured interview with some (five) traditional healers. They were interviewed individually and the approach was based on a dialogue using one of the four languages (Foulfouldé, Toupouri, Guisiga, or French) according to the traditional healers' choice. A local person was acting as a guide. The traditional healers were first informed about the objectives of the study before the beginning of the interview. The information sought were the local names of the plant, plant parts used, and medical practices such as drug preparation and administration methods for the listed diseases. The information we collected is summarized in Table 1. It comes out from this table that the treatment of lower abdominal pain consists of drinking a glass (250 mL) of the root decoction of* Entada africana* once a day for 7 consecutive days. Since the most pronounced complaint of women with endometriosis is chronic pelvic pain [17], we adopted the root decoction of* Entada africana *as extraction protocol.

The root aqueous extract of* Entada africana *was prepared following the recommendations of the traditional practitioners consulted for treating pelvic pain. Slight modifications were applied to improve the yield of extraction. One kg of fresh and clean* Entada africana* root barks was cut into small pieces, air-dried (under shade), and ground. The resulting powder (257 g) was boiled in 5 L of distilled water for 1 hour and then filtered with Whatman paper number 4. The filtrate was freeze-dried and a total dry mass of 39 g of the aqueous extract was obtained. This extract was kept at 4°C in an airtight container till use.

Three doses of this extract were administered to animals: 127.5, 255, and 510 mg/kg. After lyophilizing a little volume (250 mL) of the decoction, obtained from the traditional practitioners, a human dose of 41.1 mg/kg was extrapolated from the traditional posology. Animal equivalent dose (AED) was determined using the method described by Nair and Jacob [18]. The human dose was therefore multiplied by 6.2 to obtain an AED of 255 mg/kg. Low and high doses were obtained by dividing and multiplying 255 mg/kg by 2, respectively.

### 2.2. Animals

Young adult female Wistar rats aged 10 to 12 weeks (150 g) were obtained from the breeding facility of the Animal Physiology and Phytopharmacology Laboratory, University of Dschang (Cameroon). They were bred and kept under a standard soy-free rat diet in order to eliminate exposure to exogenous estrogenic compounds. All rats were given free access to diet and water ad libitum. Animal handling and* in vivo* experiments were carried out in conformity with the European Union on Animal Care (CEE Council 86/609) guidelines adopted by the Institutional Ethics Committee of the Cameroon Ministry of Scientific Research and Technology Innovation.

### 2.3. Study Design

Before establishing the model, estradiol valerate (Progynova® 2 mg purchased from DELPHARM (Lille, France)) at 0.5 mg/kg was orally administered to rats for 3 consecutive days to be in the estrus period, as previously described [19]. Except for the sham group (*n* = 3), other rats (*n* = 50) underwent autotransplantation surgery to establish endometriosis as described in the literature [19]. Briefly, all rats were anesthetized with an intraperitoneal administration of 10 mg/kg diazepam and 50 mg/kg ketamine. Using aseptic technique, an abdominal incision was made to expose the uterus. The left uterine horn was ligated at the cervical end using a 4-0 silk and then excised and placed in an isotonic saline. Two segments (1 cm each) of the excised horn were cut open longitudinally and attached to the intimal surface to the right side of the abdominal wall with the endometrium facing the aforesaid wall. A 4-0 absorbable thread was used to attach the graft. Sham surgeries were performed on the sham group using the same steps as the endometriosis surgeries, but no tissue was sutured to the abdominal wall. After confirming hemostasis, the midventral abdominal incision was sutured closed in layers and penicillin was injected for 3 days at 400,000 units per rat to prevent infection. After a recovery period of 28 days, rats underwent a second exploratory laparotomy to examine if models of experimental endometriosis have been successfully established. Of the 50 experimental rats, 11 did not develop any signs of endometriosis, and they were excluded from the study. The pretreatment implant volumes were calculated by measuring their dimensions (length, width, and height, in millimeters). For volume calculations, the ellipsoid volume formula (*π*/6 × length × width × height) was used [20].

Following a recovery period of 2 weeks, animals were assigned to the following groups:

(1): sham control group (*n* = 3): normal control animals treated with E_2_V (0.5 mg/kg) and the vehicle.

(2): control (*n* = 3): animals with endometriosis treated with E_2_V (0.5 mg/kg) and the vehicle.

(3): micro (*n* = 9): animals with endometriosis treated with E_2_V (0.5 mg/kg) and the reference substance, Microgynon 30 (one tablet (levonorgestrel (0.15 mg) + ethinylestradiol (0.03 mg))/rat/day). This dosage for Microgynon was adopted with reference to an experimental study using ethinylestradiol and levonorgestrel (in female rats) in the mass proportion occurring in Microgynon, to determine the mechanism whereby emotional and sexual disorders occur in hormonal contraceptive users [21].

(4): EA (*n* = 27): animals with endometriosis treated with E_2_V (0.5 mg/kg) and the root aqueous extract of* Entada africana* at the dose of 127.5 (EA 127.5, *n* = 9), 255 (EA 255, *n* = 9), or 510 (EA 510, *n* = 9) mg/kg.

In each group, E_2_V was administered daily for 14 consecutive days, 6 hours following the administration of the vehicle, Microgynon, or the root aqueous extract of* Entada africana*. Microgynon and the root aqueous extract of* Entada africana* were administered to animals for 3, 7, and 14 consecutive days as follows:

(i) For a 3-day treatment, the vehicle was administered for 11 days; Microgynon and the root aqueous extract of* Entada africana *were administered during the last 3 days.

(ii) For a 7-day treatment, the vehicle was administered for 7 days; Microgynon and the root aqueous extract of* Entada africana *were administered during the last 7 days.

(iii) For a 14-day treatment, Microgynon and the root aqueous extract of* Entada africana *were administered for 14 days.

All treatments were orally administered. On day 14, each rat received an intraperitoneal injection of oxytocin (2 U/rat) 1 h after the last administration of E_2_V to induce writhing response. Thirty minutes after observing the writhing response, animals were sacrificed under anesthesia (diazepam + ketamine). Laparotomy was performed and the volumes of ectopic foci were measured again as previously described [20]. A difference between both volumes (*V*2 − *V*1) was made to evaluate volume change and to compare the effect obtained in the control group to that obtained in treated groups. Following measurements, ectopic foci were excised and homogenated in 10 mM Tris (tris(hydroxymethy)aminomethane) buffer (0.1 g per 1 mL). Tissue homogenates were centrifugated at 3000 rpm for 10 minutes at 5°C. The resulting supernatants were stored at −20°C till use. This experiment was performed twice to ensure the accuracy of the results we obtained.

### 2.4. Biochemical Analysis

Catalase activity was estimated by the method of Sinha [22] which is based on the decomposition of H_2_O_2_ into water. The concentration of undecomposed H_2_O_2_ was evaluated using a calibration curve established from a standard solution (50 mM H_2_O_2_). Tissue catalase activity was determined as follows:

(1) $$\begin{array}{c} \text{C.A}=\frac{\Delta \text{DO}}{a\cdot t\cdot m}, \end{array}$$

where

  C.A is the catalase activity (mM of H_2_O_2_/min/mg of tissue);
  ΔDO is the absorbance of the sample − absorbance of the reagent blank;
 
*a* is the slope of the calibration curve;
 
*t* is the reaction time (1 minute);
 
*m* is the mass of the tissue collected for homogenization (mg).

Malondialdehyde (MDA) level was determined by the method of Wilbur et al. [23] which is based on the reaction with thiobarbituric acid (TBA) at 90–100°C. In the TBA test reaction, MDA or MDA-like substances and TBA react with the production of a pink pigment having an absorption maximum at 532 nm. Tissue level of MDA was determined using the following formula:

(2) $$\begin{array}{c} \left[\;\text{MDA}\;\right]=\frac{\Delta \text{DO}}{\varepsilon \cdot L\cdot m}, \end{array}$$

where

  [MDA] is the concentration of MDA (nM/mg of tissue);
  ΔDO is the absorbance of the sample − absorbance of the reagent blank;
 
*ε* is the molar extinction coefficient (1.56·10^−4^ nM^−1^ cm^−1^);
 
*L* is the path length (1 cm);
 
*m* is the mass of the tissue collected for homogenization (mg).

### 2.5. Histological Analysis

Histological analysis of the ovaries, uterus, and vagina were assessed from 5-*μ*m sections of paraffin embedded tissues. Following the hematoxylin-eosin staining, uterine and vaginal epithelial heights were assessed on microphotographs using the complete Zeiss equipment consisting of a microscope Axioskop 40 connected to a computer where the image was transferred and analysed with the MRGrab1.0 and Axio Vision 3.1 software, all provided by Zeiss (Hallbergmoos, Germany). Ovarian follicles were counted on four sections of the same ovary by two investigators at different times (with one month of interval between the first and the second observation) and the final result for each ovary represents the mean of the two observations. Luteinized unruptured follicles (LUFs) were identified by the presence of oocytes not surrounded by cumulus oophorus cells within mature antral follicles [16, 24].

### 2.6. Statistical Analysis

Data are presented as mean ± SEM. Statistical significance and the difference among groups were evaluated by one-way analysis of the variance (ANOVA) followed by Tukey test for multiple comparisons, and differences were considered statistically significant at *p* < 0.05.

## 3. Results

### 3.1. Comparison of Writhing Response among Groups

**Figure 1:**
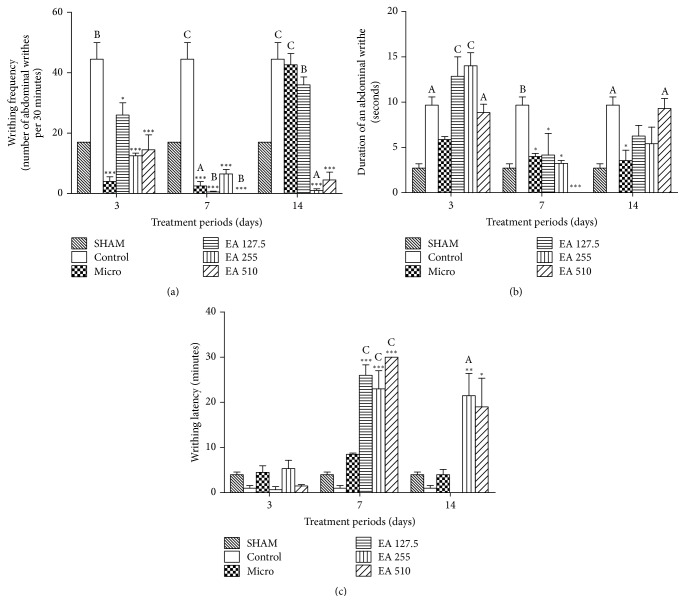
Comparison of writhing response ((a) writhing frequency; (b) duration of an abdominal writhe; (c) writhing latency) among groups. Data are presented as mean ± SEM; *n* = 3; ^A^*p* < 0.05, ^B^*p* < 0.01, and ^C^*p* < 0.001 versus SHAM; ^*∗*^*p* < 0.05, ^*∗∗*^*p* < 0.01, and ^*∗∗∗*^*p* < 0.001 versus control; treated groups were compared to SHAM and to control within the same treatment period using one-way ANOVA + Tukey's multiple comparison test; SHAM: sham operated animals; control: animals with endometriosis receiving the vehicle; micro: animals with endometriosis treated with the reference substance Microgynon; EA: animals with endometriosis receiving the aqueous extract of* Entada africana* at 127.5, 255, or 510 mg/kg BW/day.

Endometriosis induced a 2.6-fold increase in writhing frequency (*p* < 0.01 and *p* < 0.001) as compared to sham operated animals. Compared to control animals, Microgynon reduced writhing frequency by 91 and 94% (*p* < 0.001) following a 3-day and a 7-day treatment. Following a 14-day treatment writhing frequency was comparable to that of the control group. The root aqueous extract of* Entada africana* reduced writhing frequency at all tested doses following the three treatment periods. We thus noticed that following a 3-day treatment, writhing frequency decreased by 42% at 127.5 mg/kg (*p* < 0.05), 72% at 255 mg/kg (*p* < 0.001), and 67% at 510 mg/kg (*p* < 0.001). Following a 7-day treatment, writhing frequency was reduced by 99 and 85% at 127.5 and 255 mg/kg, respectively (*p* < 0.001), and completely abolished at 510 mg/kg (100% induction; *p* < 0.001). This parameter remained reduced after 14-day administration of the aqueous extract of* Entada africana *especially at doses of 255 and 510 mg/kg (*p* < 0.001) (Figure 1(a)).

The duration of an abdominal writhe was lengthened by 3.5-fold (*p* < 0.05) in control animals as compared to sham operated animals. Compared to control animals, the time of writhing was reduced with Microgynon by 39% following a 3-day treatment, 59% (*p* < 0.05) following a 7-day treatment, and 63% (*p* < 0.05) following a 14-day treatment. A similar reduction was observed with the root aqueous extract of* Entada africana* following a 7-day and a 14-day treatment. At the dose of 127.5 mg/kg, this extract decreased the duration of an abdominal writhe by 57% (*p* < 0.05) following a 7-day treatment and 35% following a 14-day treatment. At 255 mg/kg, the duration of an abdominal writhe was reduced by 66% (*p* < 0.05) following a 7-day treatment and 44% following a 14-day treatment. This parameter was completely abolished (100% reduction; *p* < 0.001) at 510 mg/kg following a 7-day treatment while no significant effect was observed following a 14-day treatment (Figure 1(b)).

Regarding writhing latency, endometriosis reduced it by 75% as compared to sham operated animals. Compared to control animals, a prominent increase in writhing latency was observed following a 7-day treatment with both Microgynon and the root aqueous extract of* Entada africana* at all tested doses. This parameter was increased by 8.5-fold following treatment with Microgynon while* Entada africana* lengthened it by 26-fold at 127.5 mg/kg (*p* < 0.001), 23-fold at 255 mg/kg, and 30-fold at 510 mg/kg (*p* < 0.001). Following a 14-day treatment, this parameter remained elevated following treatment with* Entada africana *at 255 (*p* < 0.01) and 510 (*p* < 0.05) mg/kg (Figure 1(c)).

### 3.2. Comparison of Endometrial Implant Volume, Catalase Activity, and Malondialdehyde Level

**Figure 2:**
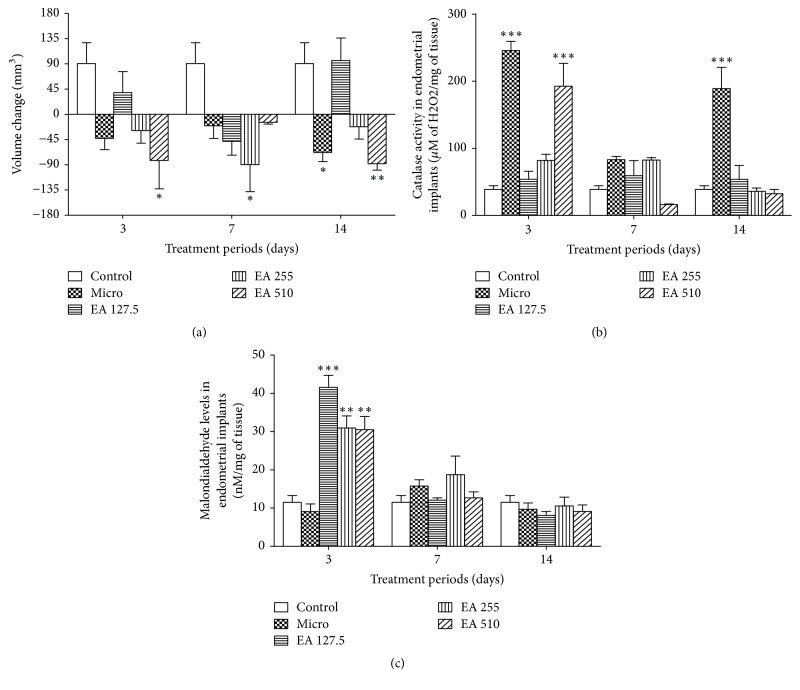
Graphical representation of volume change (a), catalase activity (b), and malondialdehyde (c) levels in endometrial implants following treatments. Data are presented as mean ± SEM; *n* = 3; ^*∗*^*p* < 0.05, ^*∗∗*^*p* < 0.01, and ^*∗∗∗*^*p* < 0.001 versus control; treated groups were compared to control within the same treatment period using one-way ANOVA + Tukey's multiple comparison test; control: animals with endometriosis receiving the vehicle; micro: animals with endometriosis treated with the reference substance Microgynon; EA: animals with endometriosis receiving the aqueous extract of* Entada africana* at 127.5, 255, or 510 mg/kg BW/day.

In the control group, implant volume was on average 22.24 ± 0.76 mm^3^ before treatment and 112.78 ± 36.41 mm^3^ 14 days following daily administration of distilled water, a volume change of 90.54 ± 37.16 mm^3^ (Figure 2(a)). This volume gain was reversed with Microgynon following the three treatment periods (147, 122, and 175% (*p* < 0.05) induction, respectively). A similar atrophy of the implants was observed with* Entada africana* following the three treatment periods at doses of 255 and 510 mg/kg (*p* < 0.05 and *p* < 0.01). The dose of 127.5 mg/kg was not able to reverse swelling of implants following the three treatment periods and rather induced a swelling of 35% lower than that of the control group following a 3-day treatment and a similar swelling following a 14-day treatment.

Microgynon increased catalase activity following the three treatment periods (*p* < 0.001) (Figure 2(b)). A 3-day treatment with the root aqueous extract of* Entada africana *increased catalase activity by 39.37% at the dose of 127.5 mg/kg. At doses of 255 and 510 mg/kg, catalase activity increased by 2- and 5-fold (*p* < 0.001), respectively. Following a 7-day and a 14-day treatment, the level of activity of catalase induced by the root aqueous extract of* Entada africana *was comparable to that observed in the control group.

Treatment with Microgynon did not significantly affect malondialdehyde (MDA) level in endometrial implants following the three treatment periods (Figure 2(c)). In contrast, the root aqueous extract of* Entada africana* significantly increased MDA levels in endometrial implants only following a 3-day treatment. This increase was 261% at 127.5 mg/kg (*p* < 0.001), 169% at 255 mg/kg (*p* < 0.01), and 165% at 510 mg/kg (*p* < 0.01). Following a 7-day and a 14-day treatment, the level of MDA was comparable and even lower than the control.

### 3.3. Ovarian Dynamic

Antral follicles were 75% higher in the ovaries of control animals than in the ovaries of sham operated animals, although this effect did not reach the level of statistical significance (Table 2). Compared to control animals, Microgynon did not significantly affect the number of antral follicles.* Entada africana *in contrast increased it by 2.7-fold at 127.5 mg/kg (*p* < 0.001), 29% at 255 mg/kg, and 2.2-fold at 510 mg/kg (*p* < 0.01) following a 3-day treatment. Antral follicles remained significantly elevated following a 7-day and a 14-day treatment at the three tested doses.

The number of corpora lutea was 2 times more elevated in control animals than in sham operated animals (*p* < 0.001). Compared to control animals, Microgynon decreased the number of corpora lutea by 53 (*p* < 0.001) and 43% (*p* < 0.05) following the three treatment periods. At 127.5 mg/kg* Entada africana* decreased the number of corpora lutea by 16 and 18% following a 3-day and a 7-day treatment, although this effect did not reach the level of statistical significance. At 255 mg/kg, an increase of 29% (*p* < 0.05) was observed following a 3-day treatment while no significant difference was noticed following a 7-day and a 14-day treatment. At 510 mg/kg, the number of corpora lutea decreased by 20% following a 3-day treatment and 32% (*p* < 0.01) following a 7-day treatment (Table 2).

Luteinized unruptured follicles (LUFs) were more elevated in control animals (6.5 per rat, *p* < 0.001) than in sham operated animals (2.7 per rat). Microgynon and* Entada africana* reduced the number of LUFs to the level of sham operated animals following the three treatment periods (Table 2).

**Figure 3:**
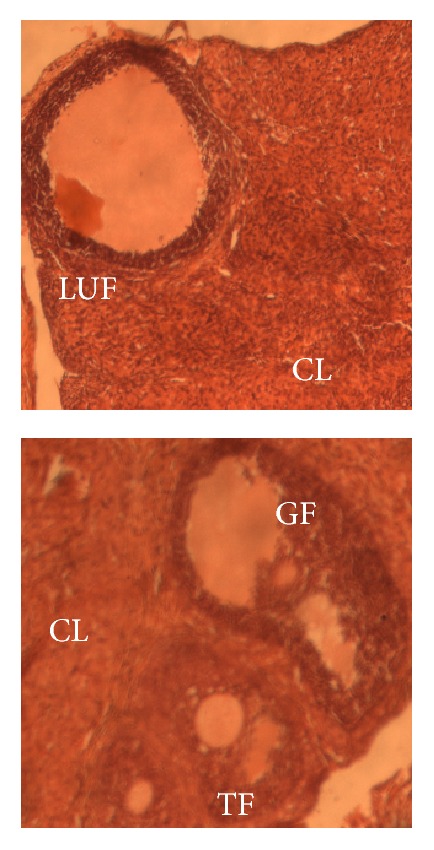
Microphotographs (×400, hematoxylin and eosin staining) of experimental rat ovaries. GF: Graafian follicle; TF: tertiary follicle; LUF: luteinized unruptured follicle; CL: corpus luteum.

Figure 3 shows microphotographs of the ovaries of experimental animals where the following follicles are identified: Graafian follicle, tertiary follicle, corpus luteum, and luteinized unruptured follicle.

### 3.4. Relative Uterine Weight, Uterine, and Vaginal Epithelial Heights

No significant difference of the relative uterine weight, as well as the uterine and the vaginal epithelial heights, was observed between sham and control groups. Similarly, the relative uterine weight and the uterine and the vaginal epithelial heights of treated groups were not significantly different from the control group (Table 3).

**Figure 4:**
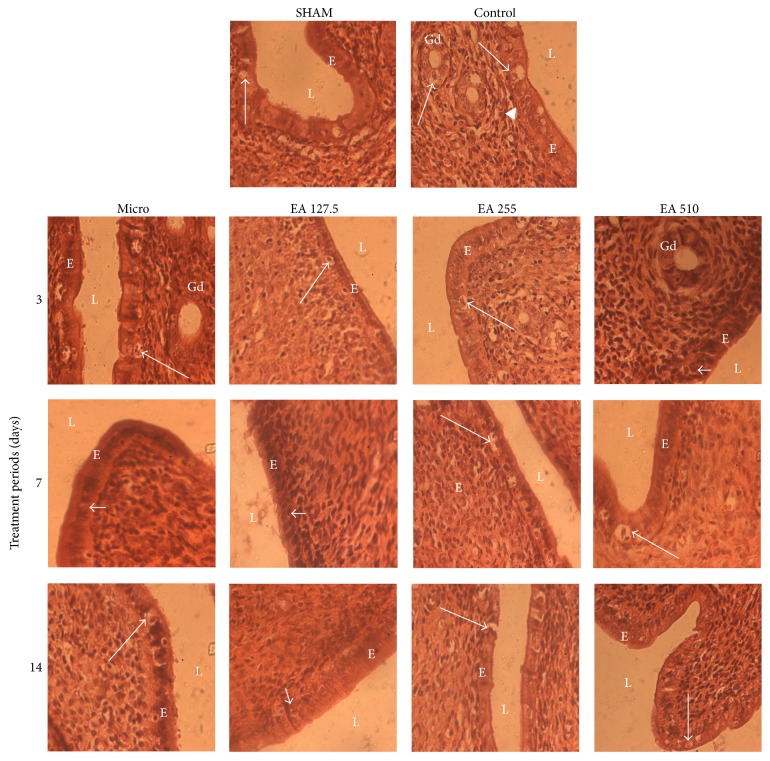
Microphotographs (×400, hematoxylin and eosin staining) of experimental rat uterine epithelium. SHAM: sham operated animals; control: animals with endometriosis receiving the vehicle; micro: animals with endometriosis treated with the reference substance Microgynon; EA: animals with endometriosis receiving the aqueous extract of* Entada africana* at 127.5, 255, or 510 mg/kg BW/day; arrow: cellular necrosis; arrow head: mitosis; E: epithelium; L: lumen.

Figure 4 shows microphotographs of the uterine epithelium. The uterus of sham operated animal was lined by a tall columnar epithelium showing cellular degeneration or necrosis. In control animals, both mitotic and necrosis figures were seen on the tall columnar uterine epithelium. In Microgynon-treated animals, the uterus was lined by a tall columnar epithelium with necrosis figure following a 3-day and a 14-day treatment. Following a 7-day treatment, few mitotic figures were seen in a tall columnar epithelium. At 127.5 mg/kg, the uterus of animals treated with the root aqueous extract of* Entada africana* was lined by a tall columnar epithelium with necrosis figures following a 3-day treatment. Following a 7-day and a 14-day treatment the tall columnar epithelium showed only few mitotic figures. At 255 mg/kg, the uterus was lined by a tall columnar epithelium with necrosis figures following the three treatment periods. At 510 mg/kg, the tall columnar uterine epithelium showed either mitotic (3-day treatment) or necrosis figures (7-day and 14-day treatment).

**Figure 5:**
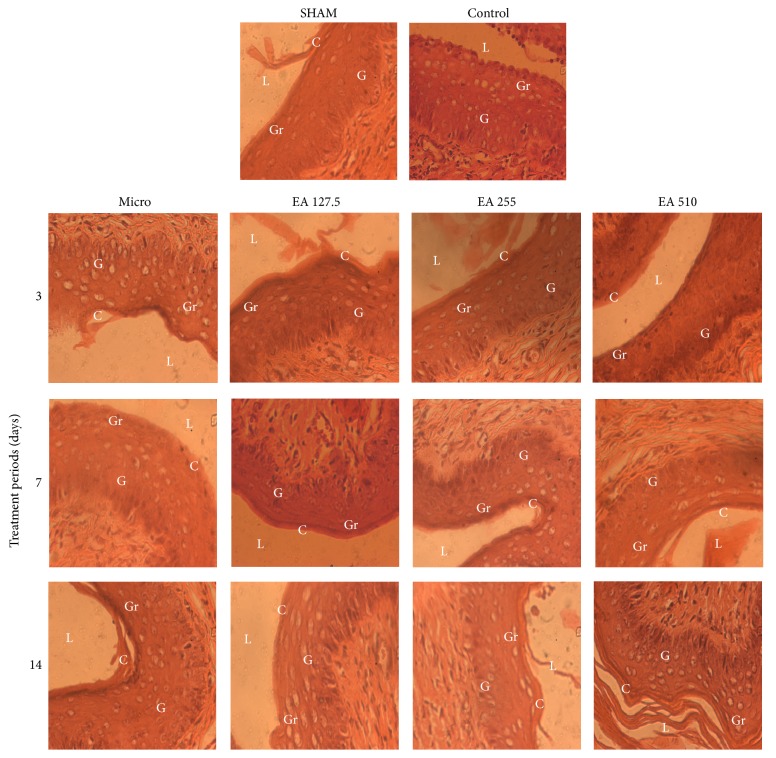
Microphotographs (×400, hematoxylin and eosin staining) of experimental rat vaginal epithelium. SHAM: sham operated animals; control: animals with endometriosis receiving the vehicle; micro: animals with endometriosis treated with the reference substance Microgynon; EA: animals with endometriosis receiving the aqueous extract of* Entada africana* at 127.5, 255, or 510 mg/kg BW/day; G: stratum germinativum; Gr: stratum granulosum; C: stratum corneum; L: lumen.

Microphotographs of the vaginal epithelium are shown in Figure 5. It comes out from this figure that the vaginal epithelium of sham operated animals was lined by a shedding cornified layer. In control animals, the vaginal epithelium was made up of a thicker stratum germinativum with a shedding stratum granulosum. In Microgynon-treated animals, the vagina was lined by a shedding cornified layer following a 3-day and a 14-day treatment. Following a 7-day treatment, the vaginal epithelium consists of a basic stratum germinativum, an intermediate stratum granulosum, and a well-attached upper cornified layer. At 127.5 mg/kg, the vaginal epithelium of animals treated with the root aqueous extract of* Entada africana* was lined by a shedding (3-day treatment) or a well-attached (7-day and 14-day treatment) cornified layer. At 255 mg/kg the vaginal epithelium was delimited by a shedding cornified layer following the three treatment periods. At 510 mg/kg the vaginal epithelium was delimited by a well-attached cornified layer following a 3-day treatment. Following a 7-day and 14-day treatment, the vaginal epithelium was delimited by a shedding cornified layer.

## 4. Discussion

Clinical symptoms of endometriosis include dysmenorrhea, nonmenstrual pelvic pain, and infertility [1]. Dysmenorrhea is defined as a cramping pain in the lower abdomen occurring just before or during menstruation [25] as a result of the interaction of uterine proinflammatory prostaglandins (especially PGF_2*α*_, released by disintegrating endometrial cells) with their receptors [26]. In rodents, dysmenorrhea is assessed as abdominal writhing responses following an intraperitoneal injection of 2 U oxytocin, one hour after the last administration of estradiol [19, 27]. Estradiol is reported to upregulate oxytocin and prostaglandin F_2*α*_ (PGF_2*α*_) receptors in rats uterus [28]. Following binding to their G protein-coupled receptors, oxytocin and PGF_2*α*_ stimulate uterine contraction through activating the phospholipase C/Ca^2+^ dependent pathway [29]. The presence of an additional endometrial tissue outside the uterine cavity during endometriosis may result in more severe dysmenorrhea. In agreement with this hypothesis, our results showed that endometriosis increased writhing frequency, decreased writhing latency, and lengthened the duration of an abdominal writhe in rats with ectopic endometrium compared to the normal control, suggesting that besides the normal uterine production of proinflammatory prostaglandins, there is additional release of these uterotonic agents by the ectopic endometrium, which would have increased dysmenorrhea.

The root aqueous extract of* Entada africana* induced Microgynon-like effects by increasing writhing latency and by reducing writhing frequency and abdominal writhing duration. These effects on dysmenorrhea were found significant at all tested doses following a 7-day treatment. These results suggest that* Entada africana* would have inhibited the signaling pathway mediated by estradiol resulting in a decreased dysmenorrhea. These analgesic effects of* Entada africana* on the dysmenorrhea rat models support the traditional use of* Entada africana* for the treatment of pelvic pain and corroborate the observations of Ezenyi et al. [9] who reported analgesic effects of* Entada africana* in acetic acid-induced nociceptive responses in mice. The increase in writhing frequency observed with Microgynon following a 14-day treatment suggests a downregulation of progesterone receptors as a result of an hyperstimulation by levonorgestrel (0.15 mg), a progestogen (a compound that induces progesterone-like effects), whose amount is 5 times more abundant than that of ethinylestradiol (0.03 mg). The literature reports that progesterone inhibits the effects of estradiol [30]. Therefore, the downregulation of progesterone receptors would have allowed the stimulation of estradiol pathway by ethinylestradiol and therefore the increase in writhing frequency.

The analgesic effects of* Entada africana* were associated with a reduced size of endometrial implants, especially following a 7-day treatment where the dose of 255 mg/kg induced a more prominent and significant effect. This result indicates a positive correlation between the progress of endometriosis and the severity of pelvic pain. To reduce endometrial implant size,* Entada africana *may act by inducing ectopic endometrium cell damage as indicated by an increased level of malondialdehyde (MDA), an end product of lipid peroxidation. Indeed, oxidative stress was associated with the development of endometriosis [31, 32]. Other studies reported that oxidative stress leads to necrosis and cell death [33–35]. Chemotherapy is known to resort to the oxidative stress mechanism which provokes cancerous cell death. Indeed, patients under chemotherapy were found to have elevated level of oxidative stress due to the destruction of cancerous cells [36]. However, a chronic oxidative stress was found to induce an inverse effect by promoting angiogenesis and tumor development [37]. A transient oxidative stress may then be crucial for the destruction of tumor cells. In agreement with this hypothesis, our results showed that the root aqueous extract of* Entada africana* increased MDA only following a 3-day treatment. This effect was reversed following a 7-day treatment and values began to be lower than those of the control group following a 14-day treatment. Previous animal studies reported decreased levels of MDA in endometrial implants following a 7-day treatment with resveratrol [20] or 5-day treatment with ozone-oxygen mixture [38]. All these treatments decreased endometrial implant volume suggesting that following treatments, there would be a transient increase in the oxidative stress of ectopic tissues that does not exceed 3 days. Results also showed that catalase activity increased in endometrial implant following a 3-day treatment. We can therefore hypothesize like Aktun et al. [38] that* Entada africana* mediated oxidative preconditioning that may improve a moderate oxidative stress which, in turn, increases antioxidant enzyme activity protecting against further growth of endometrial implants. These effects of* Entada africana* may be attributed to its content in flavonoids, especially apigenin which was reported to display antiproliferative activities [12–14].

Histological analysis of the ovaries showed elevated number of luteinized unruptured follicles (LUFs) in the ovaries of control animals as compared to sham operated animals. These results are in accordance with the literature which reports the presence of a fewer number of LUFs in the ovaries of normal animals [39]. These luteinized unruptured follicles are reported to be significantly elevated in the ovaries of animals with endometriosis as a result of the inhibition of matrix metalloproteinases by their inhibitors (tissue inhibitors of metalloproteinases), both synthetized and secreted by the eutopic and ectopic endometrium [24, 39]. The presence of more LUFs in control animals therefore supports the presence of a functional ectopic endometrium in these animals. The so-called LUF syndrome is a form of anovulation that is reported to be a subtle cause of female infertility [24, 40]. Moreover, the literature reports that despite the absence of follicular rupture and release of the oocyte, the unruptured follicles undergo luteinization under the action of LH [40] and are transformed into corpora lutea [41, 42]. The increased number of corpora lutea (which may result from the luteinization of unruptured follicles) in the ovaries of control animals suggests an accumulation due to the persistence of functional corpora lutea in these animals. Results obtained in sham operated animals with respect to antral follicles and corpora lutea suggest a contraceptive effect of the 14-day administration of E_2_V. Microgynon as well as the root aqueous extract of* Entada africana* decreased LUF number following the three treatment periods, suggesting an inhibition of the factor that promotes LUF formation, probably via the reduction of the volume of endometrial implants and, therefore, their reported ability to inhibit ovulation [24]. The increased number of antral follicles and corpora lutea (which may result from the luteinization of ovulated follicles as LUF number decreased) in animals receiving the root aqueous extract of* Entada africana* suggests that this extract would have inhibited the contraceptive effect of E_2_V and promoted follicular growth, maturation, and ovulation. Microgynon would have maintained this contraceptive effect as the number of antral follicles and corpora lutea was comparable to that of sham operated animals. Therefore, whereas endometriosis would have negatively affected ovarian dynamic by preventing ovulation, the root aqueous extract of* Entada africana* would have restored it, suggesting a beneficial effect on the fertility of animals with endometriosis.

No significant difference was observed on the relative uterus weight and the epithelial height of the uterus and the vagina in control and treated groups, suggesting that endometriosis as well as treatments did not affect both organs. However, histological analysis revealed that, unlike control animals, sham operated animals as well as treated animals were in phases of the estrous cycle controlled by oestrogens, namely, late proestrus and estrus [43]. Indeed, late proestrus is defined by a columnar uterine epithelium and a vaginal epithelium delimited by a well-attached cornified layer [43]. These figures were observed in animals treated with Microgynon following a 7-day treatment and with the root aqueous extract of* Entada africana* at 127.5 mg/kg BW following a 7-day and a 14-day treatment and at 510 mg/kg BW following a 3-day treatment. The estrus phase is in turn defined by the appearance of cellular necrosis in a tall columnar uterine epithelium while the vaginal epithelium is delimited by a shedding cornified layer [43]. Similar figures were observed in sham operated animals and Microgynon treated animals following a 3-day and a 14-day treatment, as well as in animals treated with the root aqueous extract of* Entada africana* at doses of 127.5 mg/kg BW (3-day treatment), 255 mg/kg BW (following the three treatment periods), and 510 mg/kg BW (7-day and 14-day treatment). Since a contraceptive effect of E_2_V was evoked in sham operated and Microgynon-treated animals, these results suggest that E_2_V would have induced a contraceptive effect at the central level (hypothalamus-pituitary axis) resulting in the slowing of follicular growth and maturation, as well as an agonistic effect at the peripheral level resulting in uterine and vaginal epithelial growth. In* Entada africana*-treated animals, these estrogen-controlled phases may be supported by the elevated number of antral follicles. These mature follicles were reported to release significant amount of estrogen [44]. Results obtained in control animals suggest that they were in the metestrus phase as their uterine epithelium showed both necrosis and mitotic figures while the vaginal epithelium was characterized by the absence of the cornified layer with loss of stratum granulosum [43]. This metestrus phase may be supported by the elevated number of LUFs and corpora lutea in ovaries as both were reported to produce progesterone and therefore to promote the luteal phase [44, 45].

In conclusion, endometriosis exacerbated dysmenorrhea symptoms, increased LUFs in the ovaries of experimental animals as a result of the presence of a functional ectopic endometrium, and induced them to be in the metestrus phase. The root aqueous extract of* Entada africana* decreased dysmenorrhea associated with endometriosis and this effect was positively correlated with implant volume, suggesting that the more the implant volume decreased, the lower the intensity and the duration of dysmenorrhea were. Results also suggest that* Entada africana* promoted ectopic endometrial cell damage by inducing a transient oxidative stress and prevented further growth by increasing the activity of catalase in endometrial implants. Moreover,* Entada africana* limited LUF formation in the ovaries of treated rats, supporting the observed reduction of endometrial implant volume. Animal sexual behavior would have been also modulated in favor of special periods of rat coming on heat (proestrus) and sexual desire (estrus). These preliminary findings support, at least in part, the traditional use of* Entada africana* for the treatment of pelvic pain and female infertility and suggest that the aforesaid plant could be a promising alternative option for the treatment of endometriosis.

## Figures and Tables

**Table 1:** Ethnobotanical survey of *Entada africana *in the locality of Tchabal (Cameroun Adamaoua region).

Vernacular name	Part used	Preparation mode	Diseases	Posology	Duration of treatment
(i) Padde wandu in “Foulfouldé” (ii) Wéléon in “Toupouri” (iii) Missi siriwé in “Guisiga”	Leaves	Leaf powder	Burn wounds	Poultice on the wound once a day.	Till complete healing
	Stem bark	Decoction: 1 kg of bark in 5 L of water for 2 hours.	Lower abdominal pain, hemorrhoids	A glass (250 mL) once a day.	7 days
			Eye disease	A drop into the eye.	1 day
			Malaria	Two glasses (200 mL per glass) twice a day.	4 days
		Decoction: 200 g of bark in a liter of water for 1 hour.	Diarrhea	A glass (250 mL) twice a day.	3 days
		Decoction: 250 g of bark in 500 mL of water for 1 hour.	Cough	Three glasses (200 mL) three times per day.	5 days
	Roots	Decoction: 2 g of root barks in 500 mL of water for 1 hour.	Intestinal worms	5 mL of the decoction in single catch.	1 day
		Decoction: 1 kg of root barks in 5 L of water for 1 hour.	Lower abdominal pain	A glass (250 mL) once a day.	7 days

**Table 2:** Number of antral follicles, corpus luteum, and luteinized unruptured follicles in rat ovaries.

	3-day treatment			7-day treatment			14-day treatment		
	Antral	CL/rat	LUFs/rat	Antral	CL/rat	LUFs/rat	Antral	CL/rat	LUFs/rat
	follicles/rat			follicles/rat			follicles/rat		
SHAM	1.33 ± 0.33	7.67 ± 0.89	2.67 ± 0.33	1.33 ± 0.33	7.67 ± 0.89	2.67 ± 0.33	1.33 ± 0.33	7.67 ± 0.89	2.67 ± 0.33
Control	2.33 ± 0.67	17.00 ± 1.00^c^	6.50 ± 0.29^c^	2.33 ± 0.67	17.00 ± 1.00^c^	6.50 ± 0.29^b^	2.33 ± 0.67	17.00 ± 1.00^b^	6.50 ± 0.29^c^
Micro	2.50 ± 0.29	8.00 ± 0.58^*∗∗∗*^	2.33 ± 0.67^*∗∗∗*^	2.00 ± 0.58	8.00 ± 0.58^*∗∗∗*^	2.67 ± 0.33^*∗∗*^	2.33 ± 0.67	9.67 ± 1.76^*∗*^	1.33 ± 0.67^*∗∗∗*^
EA 127.5	6.50 ± 0.29^c,*∗∗∗*^	14.33 ± 1.20^b^	2.67 ± 0.33^*∗∗∗*^	4.33 ± 0.67^a^	14.00 ± 0.58^c^	2.33 ± 0.67^*∗∗∗*^	3.67 ± 1.20	16.33 ± 1.20^a^	2.33 ± 0.33^*∗∗∗*^
EA 255	3.50 ± 0.58	22.00 ± 0.58^c,*∗*^	1.50 ± 0.29^*∗∗∗*^	4.67 ± 0.88^a^	16.67 ± 0.67^c^	2.00 ± 0.58^*∗∗∗*^	6.00 ± 0.58^a^	15.33 ± 2.19^a^	0.33 ± 0.33^a,*∗∗∗*^
EA 510	5.33 ± 0.33^c,*∗∗*^	13.67 ± 1.20^b^	3.50 ± 0.29^*∗∗*^	4.33 ± 0.33^a^	11.50 ± 0.87^a,*∗∗*^	2.67 ± 0.67^*∗∗*^	5.67 ± 1.33^a^	19.00 ± 1.16^b^	0.50 ± 0.29^a,*∗∗∗*^

Results are presented as mean ± SEM; *n* = 3; ^a^*p* < 0.05, ^b^*p* < 0.01, and ^c^*p* < 0.001 versus SHAM; ^*∗*^*p* < 0.05, ^*∗∗*^*p* < 0.01, and ^*∗∗∗*^*p* < 0.001 versus control; treated groups were compared to SHAM and to control within the same treatment period using one-way ANOVA + Tukey's multiple comparison test; SHAM: sham operated animals; control: animals with endometriosis receiving the vehicle; micro: animals with endometriosis treated with the reference substance Microgynon; EA: animals with endometriosis receiving the aqueous extract of *Entada africana* at 127.5, 255, or 510 mg/kg BW/day; CL: corpus luteum; LUFs: luteinized unruptured follicles.

**Table 3:** Relative weight of the uterus and epithelial heights of the uterus and the vagina.

	Relative uterus weight (mg/kg BW)			Uterine epithelial height (*μ*m)			Vaginal epithelial height (*μ*m)		
	3-day treatment	7-day treatment	14-day treatment	3-day treatment	7-day treatment	14-day treatment	3-day treatment	7-day treatment	14-day treatment
SHAM	1548.02 ± 26.09	1548.02 ± 26.09	1548.02 ± 26.09	1.28 ± 0.31	1.28 ± 0.31	1.28 ± 0.31	5.28 ± 1.35	5.28 ± 1.35	5.28 ± 1.35
Control	1425.53 ± 99.36	1425.53 ± 99.36	1425.53 ± 99.36	1.51 ± 0.21	1.51 ± 0.21	1.51 ± 0.21	5.31 ± 0.93	5.31 ± 0.93	5.31 ± 0.93
MICRO	1246.53 ± 186.04	1637.30 ± 240.63	1756.26 ± 101.94	2.35 ± 0.14	1.86 ± 0.15	1.69 ± 0.17	5.23 ± 0.30	7.38 ± 0.70	6.35 ± 0.22
EA 127.5	1422.60 ± 177.96	1226.79 ± 100.34	1387.03 ± 216.93	1.25 ± 0.16	1.25 ± 0.35	2.01 ± 0.41	5.48 ± 0.35	5.7 ± 0.70	6.22 ± 0.32
EA 255	1084.67 ± 68.35	1393.96 ± 98.56	1588.09 ± 18.42	2.06 ± 0.23	1.63 ± 0.13	1.14 ± 0.09	5.05 ± 0.28	4.92 ± 0.05	5.33 ± 0.84
EA 510	1385.12 ± 151.50	1458.43 ± 79.86	1297.75 ± 76.10	1.92 ± 0.33	2.03 ± 0.23	1.77 ± 0.12	4.67 ± 0.43	5.29 ± 0.30	5.31 ± 0.46

Results are presented as mean ± SEM; *n* = 3; treated groups were compared to SHAM and to control within the same treatment period using one-way ANOVA + Tukey's multiple comparison test; SHAM: sham operated animals; control: animals with endometriosis receiving the vehicle; micro: animals with endometriosis treated with the reference substance Microgynon; EA: animals with endometriosis receiving the aqueous extract of *Entada africana* at 127.5, 255, or 510 mg/kg BW/day.

## References

[1] Rahmioglu N., Montgomery G. W., Zondervan K. T. (2015). Genetics of endometriosis. *Women's Health Journal (WHJ)*.

[2] De Graaff A. A., D'hooghe T. M., Dunselman G. A. J. (2013). The significant effect of endometriosis on physical, mental and social wellbeing: Results from an international cross-sectional survey. *Human Reproduction*.

[3] Kennedy S., Bergqvist A., Chapron C. (2005). ESHRE guideline for the diagnosis and treatment of endometriosis. *Human Reproduction*.

[4] Guo S.-W. (2009). Recurrence of endometriosis and its control. *Human Reproduction Update*.

[5] Chen J. W., Du H. L., Yang J., Bai F. L., Cui J. X. (2009). The clinical study of Bushenwenyanghuayu Method in treating endometriosis. *Xian Dai Zhong Xi Yi Ji He Za Zhi*.

[6] Flower A., Liu J. P., Chen S., Lewith G., Little P. (2009). Chinese herbal medicine for endometriosis.. *Cochrane Database of Systematic Reviews (Online)*.

[7] Tchacondo T., Karou S. D., Agban A. (2012). Medicinal plants use in central Togo (Africa) with an emphasis on the timing. *Pharmacognosy Research*.

[8] Owona B. A., Njayou N. F., Laufer S. A., Schluesener H. J., Moundipa P. F. (2013). Entada africana fraction CH2Cl2/MEOH 5% inhibits inducible nitric oxide synthase and pro-inflammatory cytokines gene expression induced by lipopolysaccharide in microglia. *BMC Complementary and Alternative Medicine*.

[9] Ezenyi I. C., Ranarivelo L., Oluwakanyinsola S. A., Emeje M. (2014). Analgesic, anti-inflammatory, and heme biomineralization inhibitory properties of Entada africana ethanol leaf extract with antiplasmodial activity against Plasmodium falciparum. *Journal of Basic and Clinical Physiology and Pharmacology*.

[10] Karou S. D., Tchacondo T., Ouattara L. (2011). Antimicrobial, antiplasmodial, haemolytic and antioxidant activities of crude extracts from three selected Togolese medicinal plants. *Asian Pacific Journal of Tropical Medicine*.

[11] Germanò M. P., Certo G., D'Angelo V. (2015). Anti-angiogenic activity of Entada africana root. *Natural Product Research (Formerly Natural Product Letters)*.

[12] Guzel A., Aksit H., Elmastas M., Erenler R. (2017). Bioassay-guided isolation and identification of antioxidant flavonoids from Cyclotrichium origanifolium (Labill.) manden and scheng. *Pharmacognosy Magazine*.

[13] Yang P., Chou C., Tseng S., Hung C. (2017). Bioinformatics and in vitro experimental analyses identify the selective therapeutic potential of interferon gamma and apigenin against cervical squamous cell carcinoma and adenocarcinoma. *Oncotarget*.

[14] Yao H. M., Jia Y. P., Xue Z., Guo M., Lyu J. Y. (2017). Effects of apigenin on lipopolysaccharide induced proliferation of rat aortic vascular smooth muscle cells. *Zhonghua Xin Xue Guan Bing Za Zhi*.

[15] Tran H. N., Nguyen V. T., Kim J. A. (2017). Anti-inflammatory activities of compounds from twigs of Morus alba. *Fitoterapia*.

[16] Stilley J. A. W., Woods-Marshall R., Sutovsky M., Sutovsky P., Sharpe-Timms K. L. (2009). Reduced fecundity in female rats with surgically induced endometriosis and in their daughters: A potential role for tissue inhibitors of metalloproteinase. *Biology of Reproduction*.

[17] Amsterdam L. L., Gentry W., Jobanputra S., Wolf M., Rubin S. D., Bulun S. E. (2005). Anastrazole and oral contraceptives: a novel treatment for endometriosis. *Fertility and Sterility*.

[18] Nair A., Jacob S. (2016). A simple practice guide for dose conversion between animals and human. *Journal of Basic and Clinical Pharmacy*.

[19] Chen J., Du H., Tong R., Yang H., Ma H. (2015). Effect of Bushenwenyanghuayu decoction on nerve growth factor and bradykinin/bradykinin B1 receptor in a endometriosis dysmenorrhea mouse model. *Journal of Traditional Chinese Medicine*.

[20] Yavuz S., Aydin N. E., Celik O., Yilmaz E., Ozerol E., Tanbek K. (2014). Resveratrol successfully treats experimental endometriosis through modulation of oxidative stress and lipid peroxidation. *Journal of Cancer Research and Therapeutics*.

[21] Santoru F., Berretti R., Locci A., Porcu P., Concas A. (2014). Decreased allopregnanolone induced by hormonal contraceptives is associated with a reduction in social behavior and sexual motivation in female rats. *Psychopharmacology*.

[22] Sinha A. K. (1972). Colorimetric assay of catalase. *Analytical Biochemistry*.

[23] Wilbur K., Bernhein F., Shapiro O. (1949). Determination of lipid peroxydation. *Arch. Biochem. Biophys*.

[24] Stilley J. A. W., Birt J. A., Nagel S. C., Sutovsky M., Sutovsky P., Sharpe-Timms K. L. (2010). Neutralizing TIMP1 restores fecundity in a rat model of endometriosis and treating control rats with TIMP1 causes anomalies in ovarian function and embryo development. *Biology of Reproduction*.

[25] Harlow S. D., Ephross S. A. (1995). Epidemiology of menstruation and its relevance to women's health. *Epidemiologic Reviews*.

[26] Dawood M. Y., Khan-Dawood F. S. (2007). Clinical efficacy and differential inhibition of menstrual fluid prostaglandin F2*α* in a randomized, double-blind, crossover treatment with placebo, acetaminophen, and ibuprofen in primary dysmenorrhea. *American Journal of Obstetrics & Gynecology*.

[27] Ren X.-X., Ding X.-Y., Guo M.-W. (2011). Effects of instant electroacupuncture at the different acupoints on IP3 in the uterus tissue of dysmenorrhea model rats. *Zhongguo Zhen Jiu*.

[28] Dong Y.-L., Yallampalli C. (2000). Pregnancy and exogenous steroid treatments modulate the expression of relaxant EP2 and contractile FP receptors in the rat uterus. *Biology of Reproduction*.

[29] Arthur P., Taggart M. J., Mitchell B. F. (2007). Oxytocin and parturition: A role for increased myometrial calcium and calcium sensitization?. *Frontiers in Bioscience*.

[30] Liu Y., Tian L. B., Yang H. Y., Zhang H. P. (2017). Effects the growth of HeLa cervical cancer cells. *European Review for Medical and Pharmacological Sciences*.

[31] Grzegorz P., Diana M., Ewelina R., Aldona N., Magdalena D.-P., Jan K. (2011). Increased oxidized LDL cholesterol levels in peritoneal fluid of women with advanced-stage endometriosis. *Ginekologia Polska*.

[32] Turgut A., Ozler A., Goruk N. Y., Tunc S. Y., Evliyaoglu O., Gül T. (2013). Copper, ceruloplasmin and oxidative stress in patients with advanced-stage endometriosis. *European Review for Medical and Pharmacological Sciences*.

[33] Federico A., Dallio M., Masarone M. (2016). The epidemiology of non-alcoholic fatty liver disease and its connection with cardiovascular disease: role of endothelial dysfunction. *European Review for Medical and Pharmacological Sciences*.

[34] Dypbukt J. M., Ankarcrona M., Burkitt M. (1994). Different prooxidant levels stimulate growth, trigger apoptosis, or produce necrosis of insulin-secreting RINm5F cells: the role of intracellular polyamines. *The Journal of Biological Chemistry*.

[35] Stefan C., Nobel I., Kimland M., Lind B., Orrenius S., Slater A. F. G. (1995). Dithiocarbamates induce apoptosis in thymocytes by raising the intracellular level of redox-active copper. *The Journal of Biological Chemistry*.

[36] Todorova I., Dinev D. (2010). Comparison of the intensity of oxidative stress during chemotherapy alone and chemotherapy combined with antioxidant therapy in spontaneous mammary tumours in dogs. * Revue de Médecine Vétérinaire*.

[37] Taddei M. L., Giannoni E., Raugei G. (2012). Mitochondrial oxidative stress due to complex I dysfunction promotes fibroblast activation and melanoma cell invasiveness. *Journal of Signal Transduction*.

[38] Aktun L. H., Acet M., Atilgan R. (2016). Ozone (O3)-oxygen mixture therapy inhibits endometrial implant growth. *International Journal of Clinical and Experimental Medicine*.

[39] Moon C. E., Bertero M. C., Curry T. E. (1993). The presence of luteinized unruptured follicle syndrome and altered folliculogenesis in rats with surgically induced endometriosis. *American Journal of Obstetrics & Gynecology*.

[40] LeMaire G. S. (1987). The luteinized unruptured follicle syndrome: anovulation in disguise.. *Journal of Obstetric, Gynecologic, & Neonatal Nursing*.

[41] Ren Y., Cowan R. G., Harman R. M., Quirk S. M. (2009). Dominant activation of the hedgehog signaling pathway in the ovary alters theca development and prevents ovulation. *Molecular Endocrinology*.

[42] Tomac J., Cekinovć D., Arapović J. (2011). Biology of the corpus luteum. *Periodicum biologorum*.

[43] Westwood F. R. (2008). The Female Rat Reproductive Cycle: A Practical Histological Guide to Staging. *Toxicologic Pathology*.

[44] Marieb E. N., Hoehn K. Anatomie et Physiologie Humaines.

[45] Westfahl P. K. (1993). Comparison of luteinized unruptured follicles and corpora lutea: Steroid hormone production and response to luteolytic and luteotropic agents. *Biology of Reproduction*.

